# Validation of a moral distress instrument in nurses of primary health care^1^

**DOI:** 10.1590/1518-8345.2227.3010

**Published:** 2018-05-17

**Authors:** Priscila Orlandi Barth, Flávia Regina Souza Ramos, Edison Luiz Devos Barlem, Graziele de Lima Dalmolin, Dulcinéia Ghizoni Schneider

**Affiliations:** 2 PhD, Nursing Department, Universidade Federal de Santa Catarina, Florianópolis, SC, Brazil. Post-doctorate degree student, Programa de Pós Graduação em Enfermagem da UFSC (PEN/UFSC).; 3 PhD, Full Professor, Nursing Department, Univerisdade Federal de Santa Catarina, Florianópolis, SC, Brazil.; 4 PhD, Adjunct Professor, Nursing Department, Universidade Federal de Rio Grande, Rio Grande, SC, Brazil.; 5 PhD, Adjunct Professor, Nursing Department, Universidade Federal de Santa Maria, Santa Maria, RS, Brazil.; 6 PhD, Adjunct Professor, Nursing Department, Universidade Federal de Santa Catarina, Florianópolis, SC, Brazil.

**Keywords:** Validation Studies, Nurses, Primary Health Care, Ethics, Stress Psychological, Moral

## Abstract

**Objective:**

to validate an instrument to identify situations that trigger moral distress in relation to intensity and frequency in primary health care nurses.

**Method:**

this is a methodological study carried out with 391 nurses of primary health care, applied to the Brazilian Scale of Moral Distress in Nurses with 57 questions. Validation for primary health care was performed through expert committee evaluation, pre-test, factorial analysis, and Cronbach’s alpha.

**Results:**

there were 46 questions validated divided into six constructs: Health Policies, Working Conditions, Nurse Autonomy, Professional ethics, Disrespect to patient autonomy and Work Overload. The instrument had satisfactory internal consistency, with Cronbach’s alpha 0.98 for the instrument, and between 0.96 and 0.88 for the constructs.

**Conclusion:**

the instrument is valid and reliable to be used in the identification of the factors that trigger moral distress in primary care nurses, providing subsidies for new research in this field of professional practice.

## Introduction

Moral distress (MD) has been discussed for more than three decades since in 1984 the first concept of moral distress in nursing was presented. Identified as a psychological imbalance caused by the failure to perform an action viewed as morally correct, due to institutional barriers, managerial reluctance, lack of human and material resources, among others^1^.

In the 2000s, moral distress was related to the situation when a nurse is unable to perform an action, the psychological responses are triggered and presented in the work environment^2^. Since then, the concept has undergone changes and extensions by researchers from different parts of the world.

As a theoretical reference supporting this study, the extension of the concept proposed in Brazil was adopted, whereby moral distress is identified as a procedural phenomenon and at the same time a unique experience that integrates the ethical and moral experience of the subject. In this perspective, the moral distraction encompasses elements articulated from the ethical experience of each human being, such as the moral problem, moral uncertainty, moral sensibility, moral deliberation and moral professional ethical skills. The MD or moral suffering is considered as the interruption or failure of the process of moral deliberation and not only by its negative consequences but in its productivity or potential to propel and promote the development of moral skills, reflexivity, and resources for deliberation^3^.

A pioneer scale was developed in the North American context between 1994 and 1997^4^, to verify the triggering factors and to infer the intensity and frequency of moral distress. The Moral Distress Scale (MDS) contained 32 items on a Likert scale from 1 to 7, where 1 is never frequent/none and 7 is very frequent/very intense, as commonly used in psychometric studies, that is, to measure attitudes or behaviors, such as the case of MDS. The first version of MDS was applied to 214 hospital nurses (working in intensive and occupational units), showing moderate levels of moral distress in these professionals^4^ ^-^ ^5^.

A second MDS application containing 38 questions was performed with 106 nurses from medical and surgical units at two American hospitals to assess the intensity and frequency of moral distress. It was pointed out the cause “to work with levels of personnel that I consider to be insecure” as greater frequency and intensity and “to respond to the patient´s request for assistance to euthanasia when the patient has a poor prognosis” as lower frequency and intensity of moral distress^6^.

In Brazil, the MDS was translated and validated in its original form for the first time in 2009, containing 38 questions, and 21 questions validated by the application in 136 hospital nurses^7^. This validation obtained results similar to the application of MDS in the American scenario, with the factor of greater intensity and frequency 20 “lack of competence of the work team”^6^.

In 2012, the same author expanded his research by adapting MDS to the Brazilian context, since he realized that many situations of moral distress already observed in this scenario were not sufficiently contemplated in the original version, adding and validating another thirteen questions, where 23 questions were validated of a total of 39, with other nursing professionals, such as technicians and nursing assistants, in two hospitals in the south of Brazil. The second research also revealed the “lack of competence of the work team” with the greatest intensity and frequency of moral suffering^8^.

Since then, in several scenarios in the world context, MDS has been applied, reviewed and expanded^9^ ^-^ ^11^. Its last MDS-Revised or MDS-R version is structured with 22 items on a Likert scale from 0 to 4 for frequency and intensity, where 0 is never frequent/no intensity and 4 is very frequent/very intense^9^. However, it has its orientation to the hospital scope, and although it is broadened and validated in the Brazilian hospital scenario^7^ ^-^ ^8^, it is limited to the other scenarios of professional nurse performance.

Given the breadth and specificity of the Brazilian scenario and specifically the configuration of Primary Health Care (PHC) and the nurse’s performance in this scenario, it is imperative to investigate the moral distress in this perspective. Thus, the objective of this study was to validate a Brazilian instrument to identify the intensity and frequency of situations triggering moral distress in primary care nurses.

## Methods

This is a methodological study of adaptation and validation of the Brazilian Scale of Moral Distress in Nurses, for PHC nurses, for the inexistence of specific instruments for this purpose and scenario. The Brazilian Scale of Moral Distress in Nurses was elaborated by researchers from three federal universities in Brazil between 2014 and 2015, following the steps for their elaboration: 1) elaboration and application of a Survey with 711 nurses working in different health care scenarios in different regions of Brazil; 2) Analysis of the Survey and elaboration of initial categories to construct the questions of the instrument; 3) Carrying out a comprehensive literature review on national and international databases, matching the findings of the Survey; 4) Elaboration of the first version of the instrument; 5) Validation of the instrument by experts and pre-test; 6) Final version of the instrument containing 57 questions on a Likert scale from 0 to 6 for intensity and frequency (0 - never/none and 6 - very frequent/very intense); 7) Application of the instrument with nurses working in all contexts of Brazilian health care through an electronic form goodle.docs^12^.

The Brazilian Scale of Moral Distress in Nurses was applied in a total of 1,226 nurses from different health care settings, 391 were in the scope of PHC being the objects of this study. This study was carried out in accordance with the international recommendations for elaboration and validation of questionnaires, following eight steps^13^, including content, criterion and construct validation, described below.

In the first step, on a clear determination of what will be measured^13^, an integrative review on moral distress was performed in PHC to identify its characteristics and trigger factors in this scenario. The search was performed in the Virtual Health Library, in all its databases, from July to August 2015 through the descriptors Nursing and Primary Health Care using the term “AND” and temporal cut from 2006 for the institution of the National Policy of Primary Care (PNAB) by ordinance 648/2006. The choice of broad descriptors for the study focus is due to the inexistence of specific descriptors and the lack of knowledge about the subject, to enable to seek results in studies that interface with the phenomena.

There were 411 publications selected and 21 of them were selected according to established criteria, that is, they contain the descriptors in the abstract and/or title, in Portuguese and published from 2006. The analysis of the articles was carried out from the three stages of the Content Analysis: pre-analysis, material exploration and material interpretation^14^. In the end, it came in three main categories: Work Organization, Working Conditions, and Professional/Personal Relationships, pointing out the dynamism in the relationships among staff, among patients and with health education activities. 

The second step, elaboration of a set of items^13^ was carried out from the integrative review, in which the categories elucidated helped in the evaluation of the specific issues of moral distress in the work of the nurse in PHC. At that moment, each of the 55 questions of the original instrument was analyzed, evidencing that all were present in the literature and pointed to situations triggering moral distress in PHC. However, two gaps have been identified, one in the social vulnerabilities and the other in the educational practices under the PHC. As a result of these shortcomings, two questions were inserted that included: “Recognizing that educational actions with the patient are insufficient” and “Feeling unable to defend the patient in situations of social vulnerability”. Thus, the adaptation of the instrument contemplated the 55 original questions and two more questions elaborated from the identification of gaps on the PHC, totaling 57 questions.

In the third step, the measurement format^13^ was determined, and two Likert six-point scales were maintained, according to the original instrument, one measuring the intensity of MD ranging from 0 (none) to 6 (for very intense suffering), and the other by measuring the frequency with which MD conditions occur, ranging from 0 (never) to 6 (very common).

The fourth step consisted of a review of the set of items by specialists^12^, considering the validity of face and content. At that moment, the questions were evaluated for the language used, if they were also comprehensible by nurses of the PHC and evaluated for the representation of the content that was wanted to be analyzed. Thus, it was evaluated by an expert board composed of one expert from PHC and two from nursing ethics; and then a pre-test with 30 nurses from different Brazilian states was performed, to have acted or to be working for a minimum period of six months in the PHC as an inclusion criterion. After these validations, the instrument was considered approved to be applied in the context of PHC and in other scenarios.

The fifth step refers to the consideration of inclusion of validation items^13^, in which separately validated items can be attached to the instrument, but it was chosen to validate the items together after the final constitution of the scale. To complement the MD assessment in the PHC, some socio-demographic and labor variables were included at the beginning of the instrument, such as gender, age, country, time of action, training time, number of links, complementary training, places of work, nature of the link and workload.

In the sixth step, administration of the items in a sample^13^, the instrument was applied in a sample of the population of interest, that is, the site comprised the Brazilian PHC. Thus, all modalities of PHC teams were incorporated.

According to data from the National Register of Health Establishments of Brazil (CNES), Brazil has 50,165 health teams belonging to the PHC. The number stratified by region and the number of nurses per region are shown in Table 1.

**Table 1:** Quantitative nurses and primary care nurses in the Brazilian regions, Florianópolis, SC, Brazil, 2017

Region	Health Establishment	Nurses
Northeast	18.407	17.038
Southeast	16.502	23.602
South	7.315	9.220
North	4.460	4.634
Midwest	3.481	4.542
Total	50.165	59.036

A simple random sampling was defined for the study, based on the selection of a random sample of a population, used when a population is believed to be homogeneous, as in the case of this study^15^. Nurses with a minimum of six months’ work in the PHC were included.

However, to avoid possible biases in relation to the sample size, a minimum sample calculation was performed, as follows:

$$\frac{n={\text{X}}^{2}\cdot N\cdot P(1-P)}{d^{2}(N-1)+X^{2}\cdot P(1-P)}$$

In which:

n = sample size

X² = Chi-square value for 1 degree of freedom at the confidence level of 0.05 and equal to 3.89 (predetermined fixed value).

N = population size

P = proportion of the population to be estimated (it is assumed to be 0.50 since this proportion would provide the maximum sample size)

d = degree of precision expressed in proportion (0,05)

Considering a population of 59,036 PHC nurses, a minimum sample of 383 participants was estimated.

Emails were sent to nurses all over Brazil with information about the research and the access link. Invitations were also sent to the health secretariats of the Brazilian states and social support was used to publicize the research and form of participation. The data were collected online through the application of the instrument via Google.docs from November 2015 to April 2016. At the end of the data collection, the sample consisted of a total of 391 participants in the PHC who answered the instrument in the period of data collection.

In the seventh step, corresponding to the evaluation of items^13^ two statistical tests were carried out to guarantee to construct validity after the application of the scale in the selected sample: factorial analysis, and internal consistency analysis using Cronbach’s alpha. In the factorial analysis, the grouping of constructs of the questions by their factorial load and verification of the commonality was done, defining orthogonal rotation Varimax as a method of extraction of analysis of the main components. The formation of the constructs was established according to two criteria: the degree of association between the variables, found through factorial loads (> 500), and their conceptual association.

Cronbach’s alpha evaluated the level of reliability of the questions through the characteristics of each question in the different constructs, verifying if they were consistent with the phenomenon researched.

Finally, in the eighth and final step, optimizing the length of the scale^13^, it is possible, to exclude some item from the formed factors after factorial analysis and internal consistency to increase the reliability of the instrument. However, since all factors presented satisfactory Cronbach´s alpha values, no exclusions were made after the factorial analysis.

This research was the multicenter macro-project entitled: The process of moral distress/suffering in nurses in different health work contexts, being submitted to the Research Ethics Committees with Human Beings of the three Universities involved, with the following final opinions: 602.598-0 of 10/02/2014 (Federal University of Santa Catarina/UFSC); 602.603-0 of 01/31/2014 (Federal University of Minas Gerais/UFMG) and 511.634 of 01/17/2014 (Federal University of Rio Grande/FURG). The ethical aspects were respected according to Resolution 466/12 of the National Health Council.

## Results

Regarding the characterization of the study participants, there were 368 (93.9%) female and 23 (5.9%) male, 65 (16.6%) aged 20 to 29 years old, 150 (38.3%) from 30 to 39 years old, 99 (25.3%) from 40 to 49 years old and 77 (19.6%) with 50 or more. Regarding the training time, 7 (1.8%) had less than 1 year, 98 (25%) had from 1 to 5 years, 111 (28.3%) had from 6 to 10 years, 76 (19.4%) had 11 to 15 years, 31 (7.9%) had from 16 to 20 years and 65 (16.6%) with more than 20 years and 3 (0.7%) did not respond to this question.

Also, complementary training was also analyzed, with 53 (13.5%) not having any complementary training, 34 (8.7%) with training, 258 (65.8%) specialization, 41 (10.5%) Master´s degree, and 5 (1.3%) with Ph.Ds. Regarding the number of links, there were 280 (71.4%) with 1 link, 94 (24.04%) with 2 links and 17 (4.3%) did not respond.

Regarding the time of performance, 190 (48.5%) were working for 5 years in the service, 89 (22.7%) from 6 to 10 years, 56 (14.3%) from 11 to 15 years, 20.1%) from 16 to 20 years and 35 (8.9%) for more than 20 years, one participant (0.2%) did not respond to this question. The workload was represented by 9 (2.3%) who operated up to 20 hours, 67 (17.1%) from 21 to 30 hours, 232 (59.2%) from 31 to 40 hours and 83 (21.2 %) more than 40 hours per week. The local variables of action and type of bond correspond to 100% of the participants since the sites were all of primary health care and of a public character.

Regarding the region of action, 20 participants (5.1%) were from the North region, 66 (16.9%) from the Northeast region, 163 (41.7%) from the Southeast region, 100 (25.5%) from the South region, 24 (6.1%) from the Center-West region and 18 (4.7%) did not respond to this item. It is noted the great majority of respondents from the Southeast region. The expressive participation is explained by the fact that this region has the second largest number of teams working in the PHC, with a total of 15,637 teams, according to data from the National Registry of Health Teams. On the other hand, the Northeast has the largest number of teams of the PHC with a total of 16,903, and it was the third region with the largest participants in the study.

Regarding the construct validity, this was accomplished through the exploratory factorial analysis, with Varimax rotation with the 57 questions of the instrument (between blocks), seeking to verify the discriminant validity. The first grouping suggested 8 constructs, but with grouping difficulty due to the low factorial load and low commonality between the questions.

The process of exclusion of the questions was then initiated by the criterion of inferior commonality <0.500 in the block and lower factor load <0.500 in the constructs. At the end of the analysis, there were 11 questions excluded and 6 constructs were formed, representing 71% of the variation of the original questions, evidencing an adequate degree of data synthesis.

**Figure 1:**
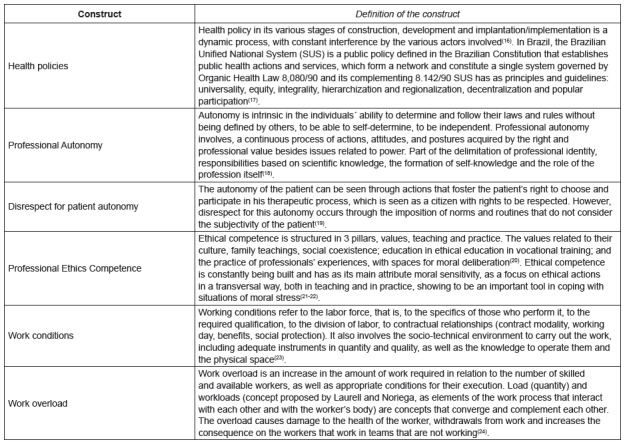
Conceptual presentation of the constructs constituted by the factorial and Cronbach´s Alpha analysis. Florianópolis, SC, Brazil, 2017

The reliability of the 6 constructs was tested by Cronbach’s Alpha applied to each construct separately and after the final instrument. Construct 1 obtained Cronbach’s Alpha of 0.96, 2 of 0.93, 3 of 0.92, 4 of 0.93, 5 of 0.94 and 6 of 0.88. The final instrument obtained Cronbach’s Alpha of 0.98. The high number of this marker indicates the reliability of the scale in the selected sample. The final version of the instrument consisted of six constructs consisting of 46 items (Table 2 and Table 3) and described conceptually (Figure 1), and the Kaiser-Meyer-Olkin test of Barllet’s sampling adequacy and sphericity was presented (Table 4 ).

**Table 2:** Exploratory Factor Analysis (Varimax rotation) of the instrument of moral distress in primary care nurses, presentation of two constructs. Florianópolis, SC, Brazil, 2017

Rotary component matrix *							
Indicators^†^		Components^‡^					
*Health policies*		C1	C2	C3	C4	C5	C6
	29. To recognize that the demands of continuity of patient/user care are not met	757	237	198	199	267	197
	30. To recognize the lack of resolution of health actions due to social problems	727	219	182	172	307	188
	32. To recognize that educational actions with the patient are insufficient	723	253	202	151	211	197
	28. To recognize that the patient´s hosting is inappropriate	699	265	250	187	218	159
	33. To experience on the humanized care practices advocated by public policies	651	307	333	202	264	156
	27. To recognize insufficient access to the service for the patient	637	218	231	172	400	104
	31. To recognize the lack of resolution of health actions due to the poor quality of care	632	209	359	240	251	194
	37. To recognize impairments to care due to inadequate integration between services/sectors	624	253	314	154	284	207
	34. To recognize routines and practices that are unsuitable for professional safety	552	265	350	182	271	212
	35. To recognize routines and practices that are inappropriate for patient safety	545	218	457	238	219	097
	44. To experience the suspension and postponement of procedures for reasons that are contrary to the needs of the patient/user	501	261	480	185	309	104
*Professional Visibility/Nurse Autonomy*							
	47. Feeling disrespected by hierarchical superiors	187	746	277	187	180	075
	17. Feeling discriminated against by other professionals	309	681	003	265	107	083
	48. To recognize ethically incorrect attitudes of managers or superiors	298	639	277	175	331	112
	38. To have their autonomy limited in the decision of specific behaviors of the nursing team	197	638	305	205	210	185
	18. Feeling devalued compared to other professionals	372	625	007	273	025	145
	41. To recognize situations of offense to the professional	245	623	311	173	251	295
	39. To experience conflicting relationships regarding the attributions of health team members	295	591	284	149	243	284
	49. Feeling pressured to agree or silence against fraud on behalf of the institution	074	560	436	177	123	075
	40. Working under pressure for insufficient time to reach goals or perform tasks	331	557	230	072	142	406
	42. To recognize situations of disrespect to the professional’s privacy	159	538	416	207	228	288

*Rotation Method: Varimax with Kaiser Standardization*. a. Converted rotation in 10 iterations.*

† Indicators, indicating the issues of the instrument with their respective numbering.

‡ Components, indicating the value (intensity) of each construct (C1 to C6).

**Table 3:** Exploratory Factorial Analysis (Varimax rotation) of the instrument of moral distress in primary care nurses, presentation of four constructs. Florianópolis-SC, Brazil, 2017.

Rotary component matrix *							
Indicadotors^†^		Components^‡^					
*Right to Health/Disrespect for patient autonomy*		C1	C2	C3	C4	C5	C6
	55. To recognize situations of disrespect to the patient’s right to confidentiality/secrecy	153	153	781	285	122	107
	54. To recognize situations of disrespect to the patient’s right to privacy/privacy	270	206	758	215	221	024
	56. To recognize situations of disrespect to patients ‘and family members’ right to information	289	209	757	208	131	110
	46. To experience care behaviors that disregard patients’ beliefs and culture	244	151	697	165	145	072
	45. To experience or participate in unnecessary care behaviors to the patient/user’s conditions/needs	270	234	634	194	148	159
	53. To recognize situations of disrespect/maltreatment by professionals in relation to the patient	385	223	599	297	181	120
*Professional Ethics Competence*							
	24.To experience the recklessness by the nurse	066	282	365	719	160	048
	22. To experience the recklessness by the doctor	251	250	161	701	142	094
	4. To work with unprepared doctors	277	090	100	656	311	324
	23. To experience the omission by the nurse	145	265	408	651	144	054
	21. To experience the omission by the doctor	340	210	224	627	133	218
	5. Work with unprepared nurses	153	074	254	590	184	459
	25. To experience the omission by professionals of other categories	174	443	328	590	233	010
	26. To experience the recklessness by professionals from other categories	152	409	314	574	244	009
*Work conditions*							
	14. To recognize that the available permanent equipment/materials are inadequate	313	175	242	192	733	196
	11. To recognize that consumer materials are insufficient	315	245	152	195	722	211
	13. To recognize that the available permanent equipment/materials are insufficient	311	248	246	152	721	259
	16. To recognize that the physical structure of the service is inadequate	300	180	161	246	691	165
	12. To recognize that consumer materials are inadequate	275	182	221	247	687	255
	15. To recognize that the physical structure of the service is insufficient	324	194	153	246	650	191
*Work overload*							
	1. To work with insufficient number of professionals to demand	256	346	098	064	259	657
	3. To experience work overload conditions	274	425	034	131	275	621
	2. Incomplete multiprofessional health team	297	253	105	137	350	620
	6. To work with unprepared nursing assistants and technicians	153	057	210	516	204	614
	7.To work with professionals from other unprepared categories	198	104	099	497	299	535

*Método de Rotação: Varimax com Normalização de Kaiser.

*a. Converted rotation in 10 iterations.*

† Indicators, with the questions of the instrument and their respective numbering.

‡ Components, with the value (intensity) of each construct (C1 to C6).

**Table 4:** Test of Kaiser-Meyer-Olkin and Bartlett*. Florianópolis - SC, Brazil, 2017.

Kaiser-Meyer-Olkin measure of sampling suitability.	.958
Bartlett sphericity test	15908.435
Approx. Chi-square df	1035
Sig.	.000

* Kaiser-Meyer-Olkin’s test of sampling suitability and Barllet’s sphericity.

Table 2 shows that the two constructs called Health Policies and Professional Visibility/Autonomy of the Nurses presented high factor loads > 500, with the highest factor load of 757 in question 29. *To recognize that the demands of continuity of patient/users are not met* in the Health Policy construct and the second with 746 in question 47. *Feeling disrespected by hierarchical superiors* of the Professional Visibility/Autonomy of the Nurse construct.

Table 3 shows the representativity of the other constructs constituted by the factorial load > 500. However, they varied between the largest load of 781 in question 55. *To recognize situations of disrespect to the patient´s right to the confidentiality/secrecy* of the construct Right to Health/Disrespect to patient´s autonomy, and the value of 657 in question 1. *Working with an insufficient number of professionals to demand* in the Work Overload construct.

Table 4 represents the reliability of the factorial analysis, when the Kaiser-Meyer-Olkin Measurement of sampling adequacy is between 0.5 and 1.0, proving that the factorial analysis was adequate. In the case of the study, this measure was close to 1.0 (0.958) proving the adequacy of the factorial analysis.

After the trustworthiness and reliability of the instrument were verified, the theoretical construction of the constructs was started, which is represented in Figure 1.

## Discussion

Moral distress in the context of primary health care, especially in the Brazilian scenario, is still a phenomenon with innumerable gaps to be explored. The validation of this instrument allows some of these gaps to be visualized and discussed to identify the factors that trigger moral distress.

The first version of the Moral Distress Scale (MDS) pointed to the validation of 3 factors, the first related to the individual responsibility of the nurse, the second not performing what would be the best for the patient, and the third to deceive, go against their values and beliefs^4^. These three factors were the same as those adopted for the application of MDS in other studies^6^.

Other researchers have reduced MDS to 21 items and applied it to physicians and nurses working in intensive care units, presenting in their analysis a difference between these professional categories in which it is appropriate to adopt treatment postures that affect the patient without any indication^25^.

Other authors have adapted MDS and/or developed other scales or surveys to measure concepts similar to moral distress in other cultures or populations^11^ ^,^ ^26^ ^-^ ^32^. The MDS was reviewed and applied in other scenarios and other professional categories, called MDS-Revised, but also with the analysis of the initial 3 factors of MDS^9^.

In the Brazilian context, in its first version of adaptation and validation of MDS, there were 4 factors identified, grouped by factorial load and conceptual coherence: 1) Denial of the role of the nurse as a patient’s lawyer; 2) Lack of competence in the work team; 3) Disrespect for patient autonomy; 4) Therapeutic Obstination (TO)^7^. In another MDS extension and validation study for all nursing categories, there were 5 constructs validated: lack of competence in the work team; disrespect for patient autonomy; insufficient working conditions; denial of the nursing role as the patient’s lawyer in the terminal; denial of the role of nursing as a patient’s lawyer^8^.

The instrument validated for primary care nurses is characterized by 6 constructs. From these constructs, “Health policies” and “Professional ethical competence” are presented as new elements in the identification of the factors that trigger moral distress.

The first construct “Health Policies” presented the largest Cronbach´s Alpha with 0.959, followed by “Working Conditions” with 0.942, “Autonomy of the Nurse” with 0.932, “Professional ethical competence” with 0.926, “Disrespect for patient´s autonomy” with 0.924 and “Workload” with 0.881.

The first construct presents questions related to patient´s access, care, continuity of care, resolution of actions, educational practices, humanized care, patient and professional safety. These questions are immersed in health policies, especially when there is a universal health system, which emphasizes equity, equality, completeness, resolve, social participation, regionalization, decentralization, and hierarchization^17^.

The second construct *Working Conditions* addresses situations that emerge from the deficiency of physical structures, equipment, materials and human resources. They are seen in different scenarios of the context of the nurse and allied to the low remuneration, to the increase of the work day and rigid hierarchy in the health team strengthen the generation of psychological suffering and poor quality of care^23^ ^-^ ^24^ ^,^ ^30^ ^-^ ^32^.

The third construct *Autonomy of the Nurse* was constituted of elements related to professional visibility, limitation in decision making, conflicts with other members of the health team for decision making and disrespect by superiors regarding conduct and professional privacy. The search for professional autonomy should be based on the delimitation of its scientifically based knowledge, as well as on the professional’s attitude towards the problems presented in their reality^33^.

The lack of autonomy and the devaluation of the nurse professional are factors that have been shown to trigger moral distress in other international settings, in which these factors are influenced by the position of other team members regarding the nurses’ conduct and the institutional rules imposed on them^34^.

The fourth construct *Professional ethical competence* was formed by situations that presented the omission, recklessness, and unpreparedness of nurses and other professional categories regarding health practices. The scenario in which these professionals work is constantly changing in terms of organizational, technological or workload, implying a constant ethical action^35^. It is in this scenario that (re) builds the ethical competence of each professional, from the moment he has problems, he assumes responsibility and builds his action based on values and beliefs, allied to his scientific knowledge and professional experience^20^ ^-^ ^21^.

The fifth construct *Disrespect for patient´s autonomy* grouped elements derived from patient´s right to privacy, choice of conduct, secrecy, access to information, devaluation of beliefs and cultures, and unnecessary conduct of care. The recognition of the singularity, individuality, completeness, legitimacy, freedom, choices of treatments and services by the patient is still incipient in the health professional’s view, participation in their therapeutic process and their right to choose, rights that must be respected for a performance of the patient^19^.

The last construct *Work Overload* has integrated imminent issues of insufficient human resources and disqualified human resources as factors that increase workload. Overworking due to such elements together with adverse working conditions tends to cause harm to the health of professionals, resulting in an increase in the lack of human resources, and consequently an increase in workload for those who remain in the service^23^ ^-^ ^24^. Evaluation and quality of care are interfered with by work overload, compromising patient safety, and consequently the trigger for experiencing moral distress^31^ ^-^ ^32^.

Thus, the 6 constructs validated 46 of the 57 questions of the instrument for the primary care sample, obeying the statistical and conceptual criteria.

## Conclusion

The results show that the instrument is able to identify the factors that trigger moral distress in nurses of primary care, providing subsidies for new research in this field of professional practice. It was possible to identify 6 constructs that exemplify the factors triggering moral distress: Health Policies, Working Conditions, Nurse Autonomy, Professional ethics, Disrespect to patient´s autonomy and Work Overload.

The validation of the instrument was supported by the analysis of factorial load and commonality greater than 0.500 and Cronbach’s Alpha <0.800 in the constructs found, considering the valid instrument for the Brazilian context of primary care, providing relevant information about the studied phenomenon. However, this study had as limitations the fact that it was the first applied and validated in the context of the primary care, which hindered to establish greater comparisons.

It is expected that the findings of this research contribute to the establishment of new discussions in the field of nursing ethics in primary care, especially in the creation of strategies that strengthen the nurse’s performance, and in the improvement of actions that promote the transformation of the actions in health.
